# Intrabronchial migration of hemostatic agent through a bronchial fistula after lung transplantation: a case report

**DOI:** 10.1186/s40792-021-01200-z

**Published:** 2021-05-10

**Authors:** Yuya Nobori, Masaaki Sato, Yasutaka Hirata, Haruo Yamauchi, Chihiro Konoeda, Kentaro Kitano, Jun Nakajima

**Affiliations:** 1 Department of Thoracic Surgery, Graduate School of Medicine, The University of Tokyo, 7-3-1 Hongo, Bunkyo-ku, Tokyo, 113-8655 Japan; 2 Department of Cardiac Surgery, Graduate School of Medicine, The University of Tokyo, 7-3-1 Hongo, Bunkyo-ku, Tokyo, 113-8655 Japan

**Keywords:** Bronchial fistula, Intrabronchial migration, Lung transplantation, Foreign body, Hemostatic agent

## Abstract

**Background:**

A bronchial fistula is a relatively rare and potentially fatal complication after lung transplantation. Thoracic surgeons and pulmonologists often face challenges when selecting treatment options. We herein report an exceptional case of intrabronchial migration of a nonabsorbable hemostatic agent, which had been placed around the pulmonary artery at the time of lung transplantation, through a bronchial fistula.

**Case presentation:**

A 61-year-old man developed respiratory distress 1 year after left single-lung transplantation for idiopathic interstitial pneumonia. Bronchoscopic examination revealed an apparent foreign body protruding from the mediastinum into the distal site of the bronchial anastomosis. The foreign body was easily removed bronchoscopically and appeared to be a hemostatic agent that had been placed during the previous lung transplantation. The patient developed a similar clinical episode and finally developed hemoptysis. Computed tomography revealed a foreign body located between the bronchus and pulmonary artery, partially protruding into the bronchial lumen. Given the possibility of a bronchopulmonary arterial fistula, surgical treatment was performed. The foreign body was located between the bronchus and left pulmonary artery and was easily removed. Multiple bronchial fistulas were found, and all were closed with direct sutures. Bypass grafting of the left pulmonary artery was then performed, initially with a homograft but eventually with an extended polytetrafluoroethylene graft. The patient was finally discharged 5 months after the surgery.

**Conclusion:**

We experienced an extremely rare case of intrabronchial migration of hemostatic agents used during the previous lung transplantation through a bronchial fistula, which were successfully managed by direct bronchial closure and bypass grafting of the left pulmonary artery.

## Background

Patients who undergo lung transplantation (LT) occasionally experience postoperative airway complications, which are associated with increased morbidity and mortality [1, 2]. The reported frequency of airway complications after LT is 1.4% [2]. A bronchial fistula is relatively rare among these complications, but has a high mortality rate and a considerable morbidity burden [1, 2]. The main risk factors for airway complications are anastomotic ischemia, surgical technique, pulmonary infection, and allograft dysfunction and rejection [1]. Appropriate management by specialized teams of thoracic surgeons, pulmonologists, and radiologists is needed because of the widely varying and complicated pathophysiology. We herein report a rare case of intrabronchial migration of a foreign body (a nonabsorbable hemostatic agent that had been placed at the time of LT) through a bronchial fistula, which was successfully managed by direct bronchial closure and bypass grafting of the left pulmonary artery.

## Case presentation

A 61-year-old man who had been diagnosed with nonspecific interstitial pneumonia underwent left LT from a brain-dead donor under peripheral cardiopulmonary bypass support due to secondary pulmonary hypertension. Because of the fragility of the recipient’s pulmonary artery (PA) and antithrombotic therapy for the cardiopulmonary bypass, refractory bleeding from the anastomosis was difficult to control during the operation even using a fibrin sealant patch (TachoSil®; Takeda Pharmaceutical Company Limited, Tokyo, Japan). Therefore, a nonabsorbable local hemostatic agent (Hydrofit®; Sanyo Chemical Industries, Kyoto, Japan) usually used in aortic surgery was applied to seal the bleeding site on the left PA. This hemostatic agent successfully stopped the bleeding; however, it later resulted in encapsulation and stenosis of the PA anastomosis, which was managed by two balloon dilation procedures [3]. The patient developed no airway complications during his hospitalization and was finally discharged 3 months postoperatively in a stable condition.

**Fig. 1:**
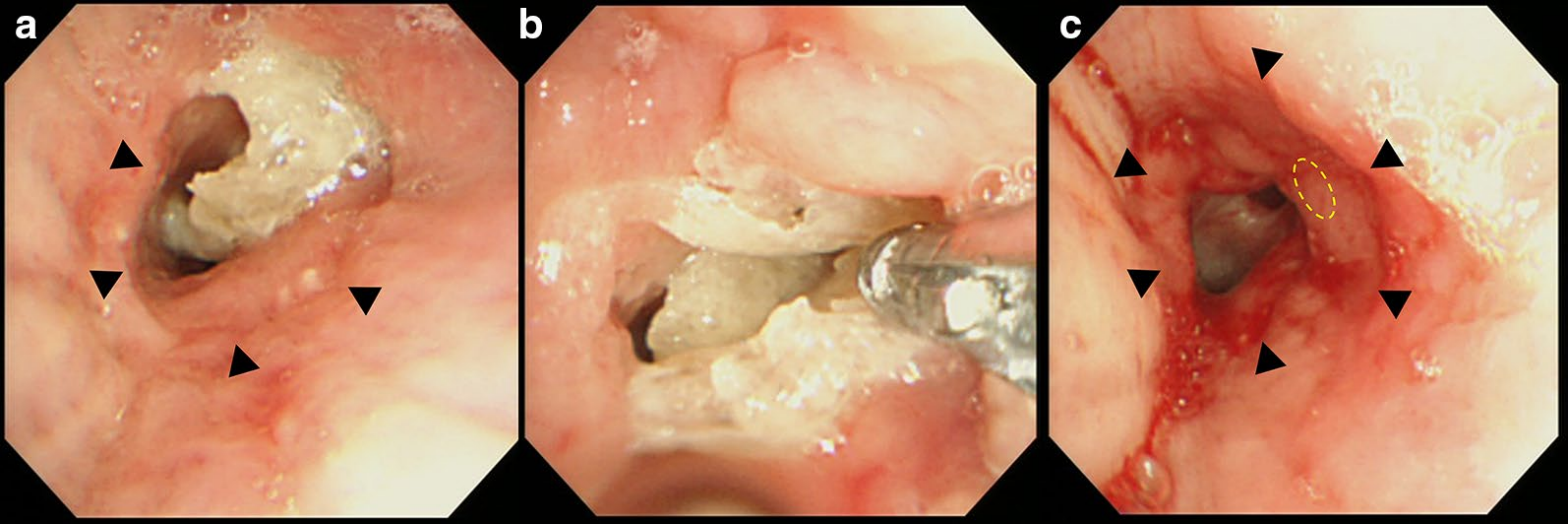
Bronchoscopic images. **a** Left main bronchus. The anastomosis of the previous transplantation (black arrowheads) and a white foreign body distal to the anastomosis are shown. **b** The foreign body was removed by bronchoscopic biopsy forceps. **c** The left main bronchus after removal of the foreign body. The granulation tissue around the fistula is indicated as yellow dotted line and the bronchial anastomosis is indicated as black arrowheads

**Fig. 2:**
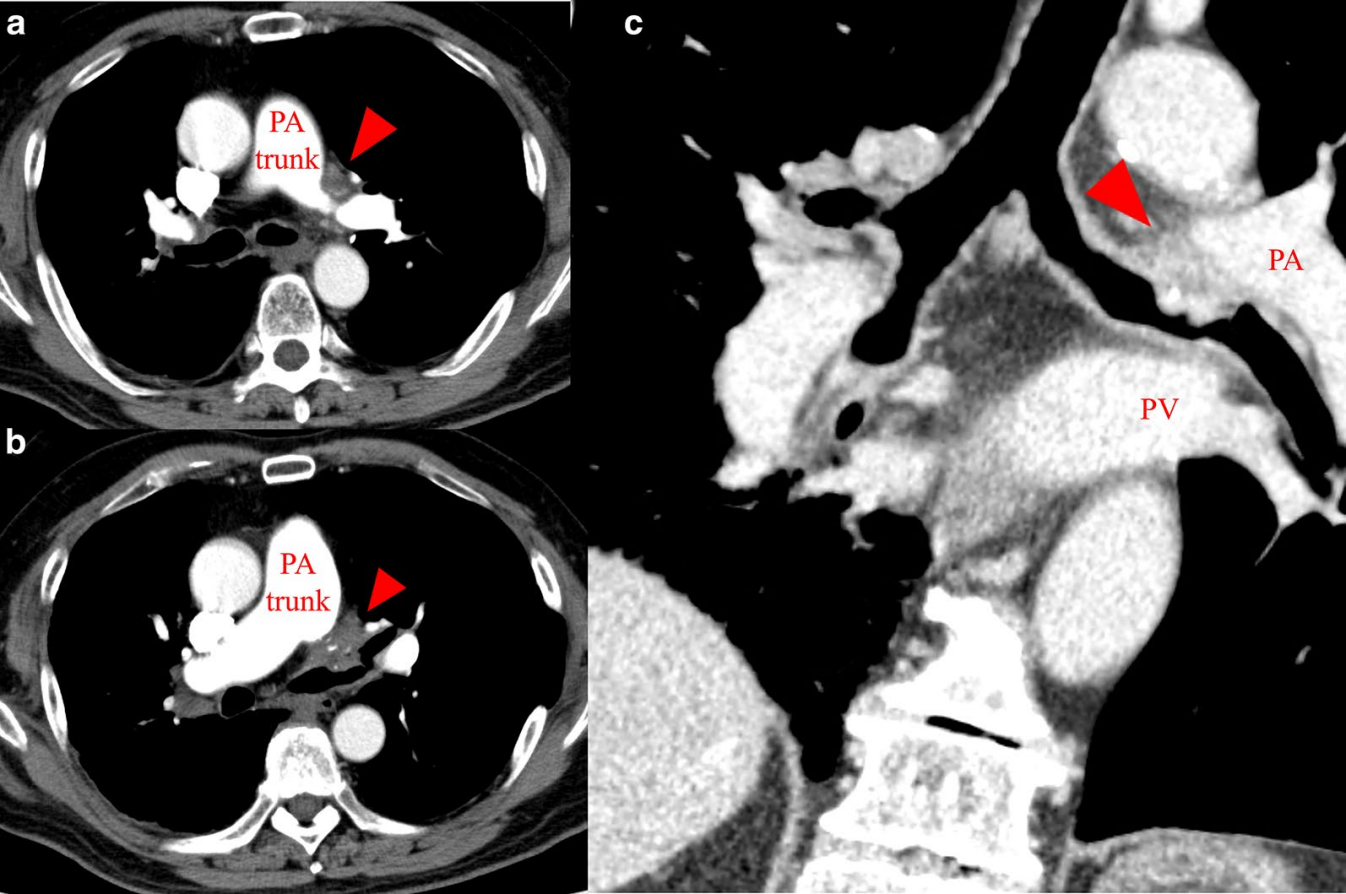
Contrast-enhanced computed tomography images. **a** Main PA stenosis surrounded by the foreign body in the mediastinum (red arrowhead). **b** The foreign body protruding to the left main bronchus (red arrowhead). **c** A coronal section showing the foreign body between the PA and left main bronchus (red arrowhead). *PA* pulmonary artery, *PV* pulmonary vein

Although he had been clinically well without any pulmonary infection or rejection after discharge, he reported a sudden decrease in his forced expiratory volume in 1 s at 1 year 2 months after LT. Bronchoscopic examination revealed what appeared to be a white foreign body protruding into the left main bronchus distal to the bronchial anastomosis (Fig. 1). It was easily removed with a bronchoscopic biopsy forceps, and the patient’s pulmonary function recovered to his baseline. This apparent foreign body appeared to be the hemostatic agent used during his LT. The pathological finding revealed eosinophilic or amphophilic amorphous structure infiltrated by inflammatory cells. The patient developed the same symptom 3 months later, and he was again successfully treated with bronchoscopic removal of a similar foreign body. One month later, however, he eventually developed hemoptysis, suggestive of a bronchopulmonary artery fistula. Contrast-enhanced chest computed tomography showed a foreign body located in the mediastinum between the left main bronchus and the left main PA, partially protruding to the left main bronchus (Fig. 2). He was urgently admitted to our hospital, and an operation for reconstruction of the bronchus and the left PA was planned.

**Fig. 3:**
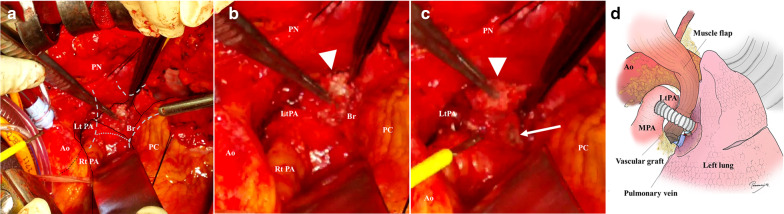
Intraoperative photos and illustrations of the hilar structures and reconstructed pulmonary artery. **a** An intraoperative picture showing the hilar structure; this picture was captured from the head camera of the operator standing on right side of the patient. The outline of left bronchus and bronchial anastomosis are illustrated as dashed lines and dotted lines, respectively. Note that the left pulmonary artery was exposed inside the pericardium and tracked peripherally toward the left hilum. Only a part of the bronchus distal to the bronchial anastomosis was exposed. **b** An enlarged picture of **a** showing a foreign body (white arrowhead) that appeared to be embedded in the bronchial wall. **c** Removal of the foreign body revealed a bronchial fistula (a white arrow). **d** An illustration of left pulmonary artery reconstructed using vascular prosthesis. *Ao* aorta, *LtPA* left pulmonary artery, *RtPA* right pulmonary artery, *Br* bronchus, *PN* phrenic nerve, *PC* pericardial tissue, *MPA* main pulmonary artery

**Fig. 4:**
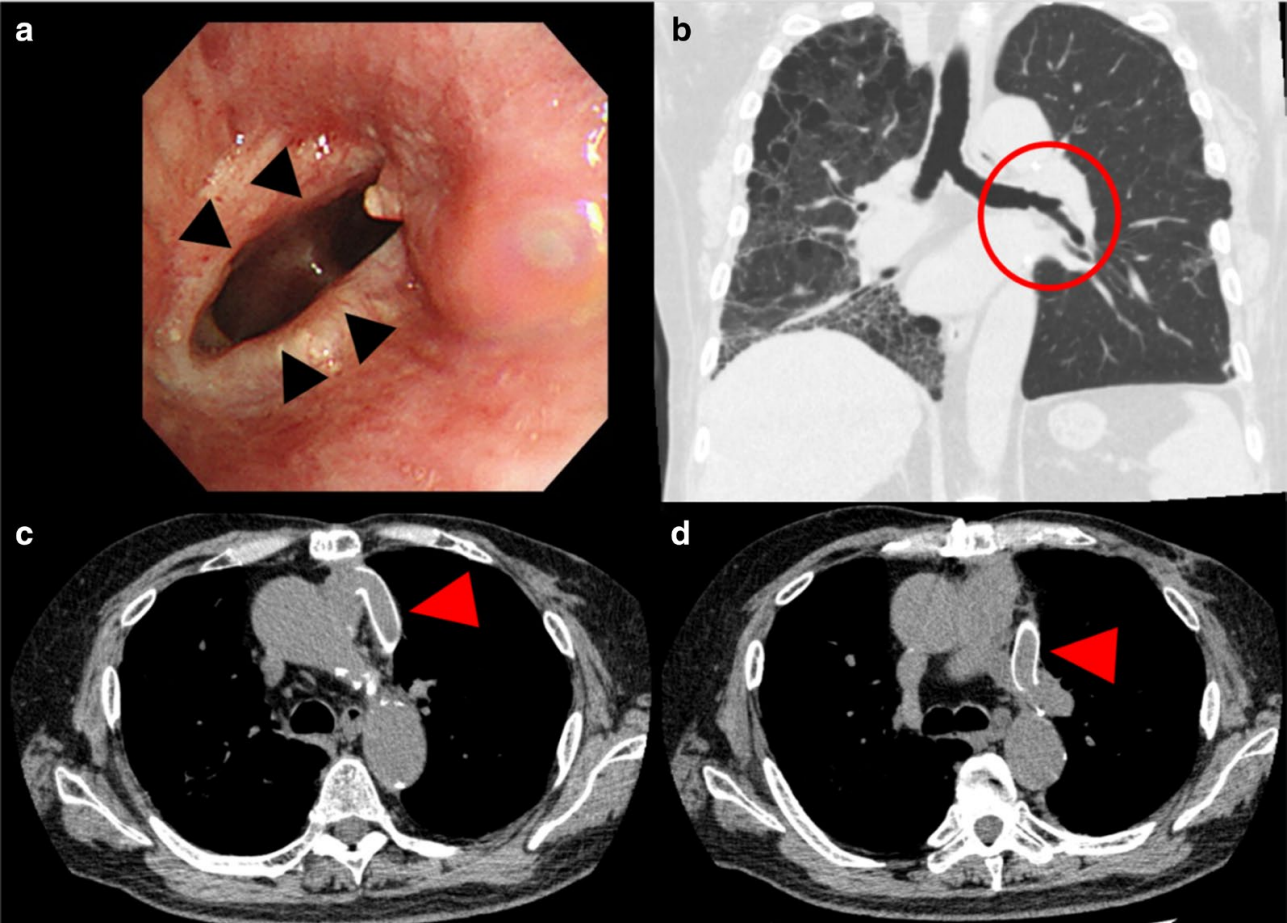
Postoperative bronchoscopic image and chest computed tomography scans 10 months after reconstructive surgery. **a** Anastomosis of the bronchus (black arrowheads). **b** Coronal section of a computed tomography scan showing the patent’s left main bronchus (red circle). **c**, **d** A vascular graft (red arrowhead) extending from the pulmonary artery trunk to the left main pulmonary artery

The operation was performed as follows. With the patient in the supine position and under general anesthesia, a median sternotomy and left third intercostal transverse incision were performed to approach the left hilum. Tight adhesion was found between the left main bronchus and the left PA anastomosis, where the foreign body was located as shown by preoperative chest computed tomography. For fear of massive hemorrhage, we started cardiopulmonary bypass from superior and inferior vena cava to ascending aorta under systemic heparinization. The foreign body was finally removed gently and thoroughly, revealing a relatively large fistula and several tiny fistulas that had spread over the bronchial wall distal to the anastomosis (Fig. 3a–c). These fistulas were closed directly with simple sutures under extracorporeal cardiopulmonary support. We initially replaced the PA with a homograft; however, because of the fragility of the homograft, we eventually exchanged the PA with a vascular graft (Triplex®; Terumo Corporation, Tokyo, Japan). A pectoralis major muscle flap was introduced between the bronchus and the left PA to separate these structures (Fig. 3d). Cardiopulmonary bypass was switched to peripheral extracorporeal membrane oxygenation from right femoral vein to right femoral artery before finishing the operation. The patient was managed in the intensive care unit for about 2 months after the surgery. Although several reoperations were needed for postoperative bleeding, thrombosis in the vascular graft, and a refractory chest wound infection, the patient was eventually discharged 5 months postoperatively. Five months later after discharge, chest computed tomography showed no abnormalities and occlusion on the left bronchus and reconstructed left pulmonary artery (Fig. 4).

## Discussion

Bronchial fistulas are categorized into three groups: bronchopleural fistulas, bronchomediastinal fistulas, and bronchovascular fistulas [1]. Our patient initially developed bronchomediastinal fistulas, which typically present with mediastinal infection. However, he had no signs of infection. This can be explained by occlusion of the fistula by the foreign body (hemostatic agents), which blocked the spread of infectious fluid into the mediastinum. Later, the patient developed hemoptysis, suggestive of a bronchovascular fistula. Because he was at high risk of massive hemorrhage and sudden death, urgent surgical treatment was unavoidable [4, 5].

Although the mechanism of bronchial fistula formation in this patient is not completely clear, we speculate that the foreign body in the mediastinum may have resulted in long-term physical friction and perforation of the bronchus due to continuous fluctuation of breathing and beating of the PA. Considering the long period of 1 year 2 months after LT and no signs of pulmonary infection and rejection, the foreign body might have gradually invaded the bronchial wall from the mediastinum. Several reports have described intrabronchial migration of a foreign body such as a peritoneal catheter, a coil, and surgical clips into the airway from the mediastinum [6–8]. This suggests the potential vulnerability of the bronchial wall to foreign bodies, although little is known about the mechanism of foreign body migration. In addition, the two balloon dilation procedures that were performed for PA stenosis after the previous surgery may have facilitated physical compression of the bronchus by the foreign body.

Hydrofit® is a nonabsorbable local hemostatic agent that is mainly applied for hemostasis of the systemic circulation (e.g., in patients with aortic anastomosis or left ventricular rupture after myocardial infarction), and its adhesive strength is reportedly very high [9, 10]. This synthetic sealant is made from hydrophilic urethane prepolymers, and these prepolymers react to water. This reaction forms strong bonds among the prepolymers, which finally change to elastic polyurethanes that exhibit firm adhesion and strong hemostasis [9]. In the present case, this sealant was tightly adhered to the anastomosis of the PA at 1 year 7 months after the previous LT. The effect of hemostasis was excellent; notably, however, such treatment has a potential risk of unpredicted complications involving the mediastinum, especially the nearby trachea and bronchus. Surgeons must be careful in the off-label use of such a sealant to avoid unexpected complications.

## Conclusion

We experienced a rare case of intrabronchial migration of a hemostatic agent implanted during the previous LT through a bronchial fistula, which was successfully managed by removal of the foreign bodies and reconstruction of the bronchus and left PA.

## Data Availability

The data used in the current study are available from the corresponding author on request.

## Abbreviations

LT: Lung transplantation
PA: Pulmonary artery

## References

[1] Mahajan AK, Folch E, Khandhar SJ, Channick CL, Santacruz JF, Mehta AC (2017). The diagnosis and management of airway complications following lung transplantation. Chest.

[2] Awori Hayanga JW, Aboagye JK, Shigemura N, Hayanga HK, Murphy E, Khaghani A (2016). Airway complications after lung transplantation: contemporary survival and outcomes. J Heart Lung Transplant.

[3] Shiraga K, Hirata Y, Saito A, Ozcelik N, Asakai H, Inuzuka R (2020). Successful angioplasties using high pressure large balloons in a patient with severe anastomotic pulmonary artery stenosis soon after single-lung transplantation. J Cardiol Cases.

[4] Cadour F, Gust L, Daviet F, Zieleskiewicz L, Dutau H, Scemama U (2020). Combined Management of a Bronchial Artery Fistula After Lung Transplantation. Ann Thorac Surg.

[5] Sourrouille I, Mordant P, Karsenti A, Dauriat G, Mal H, Leseche G (2011). Successful surgical treatment of a posttransplantation bronchovascular fistula involving the pulmonary vein. Ann Thorac Surg.

[6] Di Crescenzo V, Laperuta P, Napolitano F, Carlomagno C, Danzi M, Amato B (2013). Migration of surgical clips through a right lobectomy stump mimicking an asthmatic syndrome. BMC Surg.

[7] Kawahara T, Yanagi M, Hirano H, Arita K (2015). Intra-bronchial migration of peritoneal catheter of lumboperitoneal shunt. Surg Neurol Int.

[8] Umehara T, Aoki M, Kamimura G, Wakida K, Nagata T, Otsuka T (2017). Coil Intrabronchial Migration in an Arteriovenous Malformation Patient Treated 10 Years Ago. Ann Thorac Cardiovasc Surg.

[9] Hatori K, Kawashima T, Mori K, Kosaki S, Okamoto K, Mizoguchi T (2020). Potential utility of new surgical hemostatic film using Hydrofit((R)): a preliminary study. J Artif Organs.

[10] Ishii H, Endo H, Tsuchiya H, Inaba Y, Terakawa K, Kubota H (2018). Off-pump hemostasis for left ventricular rupture after myocardial infarction with Hydrofit((R)) and Surgicel((R)). Gen Thorac Cardiovasc Surg.

